# Prevalence and risk factors for perioperative complications of CKD patients undergoing elective hip surgery

**DOI:** 10.1186/s13018-019-1118-9

**Published:** 2019-03-20

**Authors:** Yongqing You, Yijian Zhang, Lei Qiang, Ye Sun, Junxin Zhang, Emily Bou, Moqi Yan, Kerong Dai, Muliang Ding

**Affiliations:** 1 Department of Nephrology, Affiliated Hospital of Nanjing Medical University, North District of Suzhou Municipal Hospital, Suzhou, China; 2 Southwest Jiaotong University College of Medicine, 610031, No.111, North Section,2nd Ring Road, Chengdu, Sichuan China; 3 Shanghai Key Laboratory of Orthopaedic Implants, Department of Orthopaedic Surgery, Shanghai Ninth People’s Hospital, Shanghai Jiao Tong University School of Medicine, Shanghai, 200011 China; 4 Department of Orthopedics, The First Affiliated Hospital of Soochow University, Suzhou, 215006 China; 5 Biochemistry Department, University of Waterloo, Waterloo, Canada; 6 Department of Orthopedics, The Second Xiangya Hospital, Central South University, Changsha, Hunan People’s Republic of China

**Keywords:** Chronic kidney disease, Hip surgery, Complications

## Abstract

**Purpose:**

Chronic kidney disease (CKD) is known to increase morbidity and mortality after orthopedic surgery. The purpose of this study is to investigate how CKD affects perioperative complications in hip surgery patients.

**Material and methods:**

From 2013 to 2016, a total of 230 patients (30 patients with CKD and 200 without CKD) undergoing hip surgery were enrolled in this study. Preoperative, intraoperative, and postoperative data was collected and analyzed between CKD and non-CKD patients. Logistic regression was used to evaluate the independent risk factor for postoperative complications.

**Results:**

There were significant differences in the number of people with hypertension (90.0% vs 27.3%, *P* < 0.001), diabetes (33.3% vs 8.7%, *P* = 0.01), coronary heart disease (20.0% vs 2.0%, *P* = 0.001), smoking habits (56.7% vs 22.7%, *P* = 0.016), anemia (90.0% vs 19.3%, *P* < 0.001), and low hemoglobin levels (94.1 ± 19.7 vs 121.3 ± 18.8, *P* < 0.001) between CKD and non-CKD patients before surgery. Receiving a blood transfusion was significantly more common in CKD patients (50% vs 28.5%, *P* = 0.018). Postoperatively, significant differences were detected in the average number of patients who transferred to the ICU (73.3% vs 19.3%, *P* < 0.001). Furthermore, differences were found in the quantity of hemoglobin (92.5 ± 16.8 vs 107.5 ± 18.3, *P* < 0.001) and albumin (32.4 ± 4.1 vs 34.9 ± 5.5, *P* = 0.02) measured between CKD and non-CKD patients. Logistic regression analysis indicated that diabetes, alcohol, and anemia were all independent risk factors for obtaining a blood transfusion, while age, CKD, and osteoporosis were all independent risk factors for ICU transfers.

**Conclusion:**

Compared with non-CKD patients, CKD patients were accompanied with more cardiac diseases preoperatively. In addition, CKD patients were more likely to receive a blood transfusion and transfer to the ICU after hip surgery. Preoperative anemia should be restored sufficiently to decrease the incidence of blood transfusions.

## Introduction

Chronic kidney disease (CKD) is a common and complex disease defined as eGFR (glomerular filtration rate) less than 60 ml/min per 1.73 m^2^ [1]. It is reported that the incidence of CKD doubled in the past decade, and the prevalence of end stage renal disease (ESRD) is expected to increase by 70% from 2000 to 2015 [2]. Therefore, patients with CKD need to be managed carefully during the perioperative period to avoid severe complications.

CKD patients often have disturbances in bone and mineral metabolism due to the impairment of renal function, such as secondary hyperparathyroidism, and lack of erythropoietin (EPO) synthesis and Vitamin D [3]. Previous studies indicate that patients with CKD would suffer a higher occurrence of related complications than patients with normal renal function after orthopedic surgery [4, 5]. Ackland et al. [6] reported that CKD would increase the risk of morbidity in elective orthopedic surgery, and that preoperative glomerular filtration rate (GFR) is correlated with the occurrence of postoperative complications. Deegan et al. [7] reported that end stage renal disease would increase the risk of perioperative complications in patients undergoing total hip arthroplasty (THA) and total knee arthroplasty (TKA), and that the severity of CKD is related to the mortality rate. Kuo et al. [8] concluded that CKD would reflect an increased risk for obtaining fracture nonunion and subsequent surgical revision for an intracapsular hip fracture. However, few studies have focused on the effect of CKD on perioperative complications in patients undergoing hip surgery.

In this study, we conducted a retrospective study aiming to investigate whether CKD would affect morbidities and complications after hip surgery.

## Material and methods

### Patients

From February 2013 to December 2016, a total of 230 patients who underwent hip surgery in our institution were enrolled in this study. Among the CKD patients (14 males and 16 females, mean age 73.0), 7 underwent total hip arthroplasty, 12 underwent artificial femoral head replacement, and 11 underwent internal fixation. Among the non-CKD patients (82 males and 118 females, mean age 69.2), 41 underwent total hip arthroplasty, 99 patients underwent artificial femoral hip replacement, and 60 underwent internal fixation. We confirmed that all methods were carried out in accordance with relevant guidelines and regulations. Furthermore, this study was approved by the First Affiliated Hospital of Soochow University and informed consent was obtained from all subjects.

### Inclusion and exclusion criteria

Patients with hip surgery including total hip arthroplasty, artificial femoral head replacement, and internal fixation for femoral neck and intertrochanteric fracture were enrolled in this study. Patients younger than the age of 18 patients that have had combined operations, and/or patients with incomplete information were excluded.

### Recorded data

Before surgery, we compared age, sex, hypertension, diabetes, congestive heart failure, coronary heart disease, cerebral infarction, smoking habits, alcohol consumption, osteoporosis, hepatitis, hyperlipidemia, anemia, and hemoglobin, creatinine, and albumin levels between CKD and non-CKD patients. Intraoperative parameters including operation time, blood transfusion, and surgical methods were also compared. Incidence of urinary tract infection, fever, ICU transfer, deep vein thrombosis, hospital transfer, and levels of hemoglobin, creatinine, and albumin were compared between CKD and non-CKD patients postoperatively.

### Statistical analysis

In this study, parametric data was expressed as a mean ± deviation. Continuous variables were compared using independent *t* test, and categorical variables were compared using Chi-square test. SPSS (SPSS Inc., Chicago, IL, USA) was used for data analysis. To examine the independent effect of variables on outcomes, a multiple logistic regression model was constructed. A *P* value less than 0.05 was considered significantly different.

## Results

All 230 patients received hip surgery successfully, such that no patients had fatal complications after surgery. Preoperative demographic data which included age, sex, congestive heart failure, alcohol, osteoporosis, hepatitis, hyperlipidemia, and albumin showed no significant differences between CKD and non-CKD patients (Table 1). There were significant differences in the number of people with hypertension (90% vs 27.3%, *P* < 0.001), diabetes (33.3% vs 8.7%, *P* = 0.01), coronary heart disease (20% vs 2%, *P* = 0.001), smoking habits (56.7% vs 22.7%, *P* = 0.016), preoperative anemia (90% vs 19.3%, P < 0.001), and preoperative hemoglobin (94.1 ± 19.7 vs 121.3 ± 18.8, P < 0.001). There was no significant difference in operation time and surgical methods between CKD and non-CKD patients; however, the fraction of patients who received a blood transfusion was significantly higher in CKD patients (Table 2).

**Table 1:** Comparison of demographic data between CKD group and control group

Variables	CKD group (*n* = 30)	Control group (*n* = 200)	*P* value
Age	73.0 ± 11.1	69.2 ± 15.7	0.10
Sex (male/female)	14/16	82/118	0.56
HTN (%)	90.0	27.3	< 0.001*
Diabetes (%)	33.3	8.7	0.01*
CHF (%)	3.3	0	0.13
CHD (%)	20.0	2.0	0.001*
CI (%)	6.7	4.0	0.99
Patients who smoke (%)	56.7	22.7	0.016*
Alcohol consumption (%)	36.7	21.7	0.65
Osteoporosis (%)	23.3	16.0	0.94
Hepatitis (%)	0	0.3	1.0
HLD (%)	6.7	1.0	0.13
Anemia (%)	90.0	19.3	< 0.001*
Pre-Hb	94.1 ± 19.7	121.3 ± 18.8	< 0.001*
Pre-Albumin	37.3 ± 4.5	38.5 ± 4.4	0.16
Pre-Cr	458.2 ± 282.8	64.3 ± 21.8	< 0.001*

*Abbreviation*: *HTN* hypertension, *CHF* congestive heart failure, *CHD* coronary heart disease, *CI* cerebral infarction, *HLD* hyperlipidemia, *Hb* hemoglobin, *Cr* creatinine

*P < 0.05

Values for pre-Hb, pre-Albumin, and pre-Cr are represented as average concentration (g/L)

**Table 2:** Comparison of intraoperative parameters between CKD group and control group

Variables	CKD group	Control group	*P* value
Operation time	81.6 ± 44.8	89.0 ± 40.8	0.36
Blood transfusion	15/15	57/143	0.018*
Surgical methods			0.62
THA	7	41	
AFHR	12	99	
IF	11	60	

*Abbreviation*: *THA* total hip arthroplasty, *AFHR* artificial femoral head replacement, *IFCR* internal fixation

*P < 0.05

Postoperative complication analysis indicated that there were significant differences in the percentage of ICU transfers (73.3% vs 19.3%, *P* < 0.001), levels of postoperative hemoglobin (92.5 ± 16.8 vs 107.5 ± 18.3, *P* < 0.001), and levels of postoperative albumin (32.4 ± 4.1 vs 34.9 ± 5.5, *P* < 0.02) between CKD and non-CKD patients (Table 3). In subgroup analysis, we found that patients received dialysis treatment had a higher proportion of blood transfusion and higher value of postoperative creatinine compared with non-dialysis CKD patients (Table 4). Logistic regression analysis indicated that diabetes, alcohol, and anemia were all independent risk factors for acquiring a blood transfusion (*P* < 0.05) (Table 5), while age (greater than 60 years), CKD, and osteoporosis were all independent risk factors for an ICU transfer (*P* < 0.05) (Table 6).

**Table 3:** Comparison of postoperative complications between CKD group and control group

Variables	CKD group	Control group	*P* value
UTI (%)	0	0.7	0.99
Fever (%)	20.0	13.0	0.95
ICU (%)	73.3	19.3	< 0.001
DVT (%)	0	0	1.0
Post-Hb	92.5 ± 16.8	107.5 ± 18.3	< 0.001*
Post-Albumin	32.4 ± 4.1	34.9 ± 5.5	0.02*
Post-Cr	440.7 ± 266.0	59.8 ± 20.9	< 0.001
Hospital stay	14.5 ± 4.2	13.5 ± 6.0	0.93

*Abbreviation*: *UTI* urinary tract infection, *ICU* intensive care unit, *DVT* deep vein thrombosis, *Hb* hemoglobin, *Cr* creatinine

*P < 0.05

**Table 4:** Sub-comparison between CKD patients with or without dialysis

Variables	Non-dialysis CKD group (*n* = 22)	Dialysis CKD group (*n* = 8)	*P* value
Operation time	88.3 ± 49.0	63.1 ± 23.8	0.18
Blood transfusion	8/14	7/1	0.035*
UTI (%)	0	0	1.0
Fever (%)	13.6	37.5	0.3
ICU (%)	72.7	75	1.0
DVT (%)	0	0	1.0
Post-Hb	92.8 ± 12.0	91.6 ± 27.2	0.91
Post-Albumin	32.1 ± 4.6	33.3 ± 2.3	0.36
Post-Cr	354.3 ± 218.4	678.5 ± 248.9	0.002*
Hospital stays	13.9 ± 3.6	16.1 ± 5.5	0.31

*P < 0.05

**Table 5:** Risk factors for intraoperative transfusion stay using multivariate logistic regression

Variables	Coefficient	OR (95% CI)	*P* value
Diabetes	0.91	2.49 (1.17 to 5.29)	0.018*
Alcohol	0.62	1.85 (1.00 to 3.41)	0.048*
HLD	2.09	8.11 (0.84 to 78.28)	0.07
Anemia	0.80	2.22 (1.23 to 4.03)	0.008*

*Abbreviation*: *HLD* hyperlipidemia

*P < 0.05

**Table 6:** Risk factors for intraoperative ICU transfer using multivariate logistic regression

Variables	Coefficient	OR (95% CI)	*P* value
Age (> 60)	2.58	13.21 (3.81 to 45.82)	< 0.001*
CKD	2.03	7.60 (2.82 to 20.52)	< 0.001*
Osteoporosis	0.97	2.65 (1.32 to 5.30)	0.006*

*Abbreviation*: *CKD* chronic kidney disease

*P < 0.05

## Discussion

In the present study, CKD patients showed more cardiac diseases and diabetes preoperatively. Furthermore, CKD patients had a higher risk of receiving a blood transfusion and being transferred to the ICU. It was known that patients with CKD were always accompanied with several comorbidities including cardiac, pulmonary, and liver disease preoperatively because the kidney plays an important role in regulating homeostasis. Therefore, it was difficult to manage these patients during the perioperative period. In a retrospective study by Kuo et al. [9], patients with CKD had significantly higher occurrences of cardiovascular diseases, diabetes, and gout before total knee arthroplasty. This association between CKD and other comorbidities was also verified in another study, which enrolled 13,844 patients with a 2-year follow-up [10]. In this present study, there were significant differences in the prevalence of hypertension, diabetes, coronary heart disease, smoking habits, and anemia between CKD and non-CKD patients. In addition, the impairment of renal function and related comorbidities would increase the risk of the operation itself and increase morbidity and mortality after orthopedic surgery. De la Garza Ramos et al. [11] reported that CKD or end-stage renal disease would increase the incidence of postoperative major complications for patients undergoing anterior cervical fusion, and that CKD patients had a 15-fold longer hospital stay. Puvanesarajah et al. [12] reported that CKD patients older than 65 years of age had a higher risk of experiencing major medical complications within 3 months of surgery, and that they had an increased mortality rate by a factor of 2.8 within 12 months after one or two level lumbar fusion surgery. Bains et al. [13] reported that compared with normal renal function dialysis-independent, CKD patients had a 1.78-fold increase in overall mortality rate, while dialysis-dependent CKD patients had a 3.98-fold increase in overall mortality rate after spine surgery. This indicated that preoperative renal disease as an independent risk factor for mortality after surgery.

In this study, there was no significant difference in operation time and surgical methods between CKD and non-CKD patients. However, 50% of CKD patients received an intraoperative blood transfusion compared to 28.5% in non-CKD patients (*P* = 0.018). In addition, from subgroup analysis, we found that CKD patients who received dialysis treatment had a higher postoperative creatinine level and higher rates of blood transfusion than non-dialysis CKD patients. This result indicated dialysis may have a negative influence on perioperative complications. Augustin et al. [14] reported that patients with GFR, less than 30 ml/min/1.73 m^2^, were at greater odds of receiving a blood transfusion in the hospital (OR 4.42, *P* < 0.001), meanwhile CKD, operation time, and anemia were all independent risk factors for obtaining a blood transfusion (*P* < 0.05). Tan et al. [15] also reported that CKD patients (> stage 2) showed an increased likelihood of receiving a blood transfusion. This can be seen in the linear relationship obtained between eGFR and blood transfusion. Unfortunately, in logistic regression analysis, not CKD but diabetes, alcohol, and anemia proved to be independent risk factors for blood transfusion. This phenomenon might attribute to the fact that some CKD patients did not show a severe lack in hemoglobin concentration, while some non-CKD patients lost large amounts of blood due to trauma or other systematic diseases. Moreover, a previous study demonstrated that CKD patients usually had iron-deficient anemia rather than erythropoietin deficiency, and hemoglobin levels could be improved by correcting the iron deficiency effectively. In contrast, anemia or preoperative hemoglobin was a direct indicator that strongly correlated with patient blood transfusions [16]. Graves et al. [17] also reported that CKD alone without anemia did not predispose an increased risk of obtaining a blood transfusion in patients with mild-to-moderate CKD.

CKD patients were likely to have several comorbidities after surgery, which contribute to a slow and difficult recovery. Some patients with complicated comorbidities needed to be transferred to an ICU for subsequent treatment. Adogwa et al. [18] reported that CKD patients had a higher incidence of being transferred to an ICU (52.9% vs 29.4%, *P* = 0.04) than non-CKD patients after lumbar decompression and fusion. Porter et al. [19] reported that an impairment in kidney function would increase the length of critical care stay, and therefore the severity of kidney impairment was related to the duration of critical care stay. In our study, there was a significant difference in the number of ICU transfers between CKD and non-CKD patients (*P* < 0.001). Furthermore, logistic regression analysis indicated that CKD was an independent risk factor for ICU transfer (OR 7.60, *P* < 0.001). We suspect that in CKD patients, the combination of increased mobility, higher mortality [20], and increased burden to the kidney resulted from surgery. The stress from surgery might be responsible for the higher rate of ICU transfer after hip surgery. Furthermore, CKD was not the only risk factor for ICU transfer, our results also showed that patients older than 60 and patients with osteoporosis were both independent risk factors. This indicated that anti-osteoporosis treatment was also important for these patients.

### Limitation

There were some limitations in our study. First, our study was a retrospective study and the cohort size was not very large. Second, the data related to CKD were not completely sufficient. Third, our study was only based on an inpatient database which was unavailable for 30- or 90-day outcomes. Fourth, this study lacks enough data of specific etiology for CKD, which may be hard to conduct the subgroup analysis. Further prospective, larger sample sizes, and more sufficient data about CKD in orthopedic surgery will be conducted in the future.

## Conclusion

CKD patients showed higher occurrences of preoperative cardiac disease compared with non-CKD patients. CKD patients had a higher risk of receiving a blood transfusion and being transferred to the ICU after hip surgery. Preoperative anemia was an independent risk factor for obtaining a blood transfusion and, therefore, should be focused on during the perioperative period.

## Abbreviation

CKD: Chronic kidney disease
ICU: Intensive care unit
